# The effect of rare alleles on estimated genomic relationships from whole genome sequence data

**DOI:** 10.1186/s12863-015-0185-0

**Published:** 2015-03-12

**Authors:** Sonia E Eynard, Jack J Windig, Grégoire Leroy, Rianne van Binsbergen, Mario PL Calus

**Affiliations:** Animal Breeding and Genomics Centre, Wageningen UR Livestock Research, P.O. Box 338, Wageningen, 6700 AH The Netherlands; AgroParisTech, UMR 1313 Génétique Animale et Biologie Intégrative, 16 rue Claude Bernard, Paris 05, 75231 France; INRA, UMR 1313 Génétique Animale et Biologie Intégrative, Jouy-en-Josas, 78350 France; Centre for Genetic Resources the Netherlands, Wageningen UR, P.O. Box 16, Wageningen, 6700 AA The Netherlands; Biometris, Wageningen UR, P.O. Box 16, Wageningen, 6700 AA The Netherlands

**Keywords:** Whole genome sequence, Additive genetic relationship, Rare variants, Minor allele frequency, Inbreeding

## Abstract

**Background:**

Relationships between individuals and inbreeding coefficients are commonly used for breeding decisions, but may be affected by the type of data used for their estimation. The proportion of variants with low Minor Allele Frequency (MAF) is larger in whole genome sequence (WGS) data compared to Single Nucleotide Polymorphism (SNP) chips. Therefore, WGS data provide true relationships between individuals and may influence breeding decisions and prioritisation for conservation of genetic diversity in livestock. This study identifies differences between relationships and inbreeding coefficients estimated using pedigree, SNP or WGS data for 118 Holstein bulls from the 1000 Bull genomes project. To determine the impact of rare alleles on the estimates we compared three scenarios of MAF restrictions: variants with a MAF higher than 5%, variants with a MAF higher than 1% and variants with a MAF between 1% and 5%.

**Results:**

We observed significant differences between estimated relationships and, although less significantly, inbreeding coefficients from pedigree, SNP or WGS data, and between MAF restriction scenarios. Computed correlations between pedigree and genomic relationships, within groups with similar relationships, ranged from negative to moderate for both estimated relationships and inbreeding coefficients, but were high between estimates from SNP and WGS (0.49 to 0.99). Estimated relationships from genomic information exhibited higher variation than from pedigree. Inbreeding coefficients analysis showed that more complete pedigree records lead to higher correlation between inbreeding coefficients from pedigree and genomic data. Finally, estimates and correlations between additive genetic (**A**) and genomic (**G**) relationship matrices were lower, and variances of the relationships were larger when accounting for allele frequencies than without accounting for allele frequencies.

**Conclusions:**

Using pedigree data or genomic information, and including or excluding variants with a MAF below 5% showed significant differences in relationship and inbreeding coefficient estimates. Estimated relationships and inbreeding coefficients are the basis for selection decisions. Therefore, it can be expected that using WGS instead of SNP can affect selection decision. Inclusion of rare variants will give access to the variation they carry, which is of interest for conservation of genetic diversity.

**Electronic supplementary material:**

The online version of this article (doi:10.1186/s12863-015-0185-0) contains supplementary material, which is available to authorized users.

## Background

The use of sequence data has increased considerably in the past few years and is expected to further expand due to technological improvements and a reduction in costs for whole genome sequencing [1,2]. While Single Nucleotide Polymorphism (SNP) chips, recently used in selection strategies, contain only a subset of the polymorphic variants available in a species, whole genome sequence (WGS) data provide access to complete information on all the variants of an individual. Most of the low Minor Allele Frequency (MAF) variants are only accessible through whole genome sequence data. Therefore, WGS data are expected to yield better estimators of the true relationships between individuals by accounting for all the genetic variation.

Breeding decisions are partly based on estimated relationships and inbreeding coefficients analysis of the population from which breeding individuals will be selected. Pedigree, SNP chips or WGS data can be used to estimate these coefficients. Traditional pedigree records have been used in selection strategies for about 30 years and SNP data have proven their efficiency in the last decade [2]. Nevertheless, both pedigree and SNP chips may lead to sub-optimal selection decisions, as pedigree is generally based on partial genealogic records and SNP data present ascertainment bias, due to the criteria used for the chip assembly [3,4]. As suggested in a review paper by Henryon et al. [5], even though selection has been conducted based on genomic information for some years, the utilisation of pedigree and SNP chip data for the estimation of relationships and genetic variation can still be further optimised. This may be achieved by the use of whole genome sequence (WGS) data. One of the major advantages of WGS, is that it not only captures all common variants in the genome, but accesses the many variants with rare alleles not covered by SNP chips as well. In addition, the increasing availability of WGS data coincides with reinforced attention for the development of long-term selection strategies and the impact of short versus long-term strategies on the genetic diversity of livestock species [6]. This may open up new possibilities for the optimisation of animal selection in the long-term perspective and for the prioritisation of animal selection in a conservation focused context [7-9].

Even though whole genome sequence data are becoming increasingly abundant, an important question is if it is worth investing in such a technique, or whether traditional data, i.e. a limited number of SNP variants and pedigree, are sufficient for long-term selection strategies and prioritisation of animals for genetic diversity conservation [10]. Thus, several major questions need to be addressed. Are relationships computed from WGS data, including information from rare alleles, different from those computed from pedigree and SNP data? Will the use of this type of data help to further develop selection strategies that optimise the long-term improvement and genetic diversity conservation of livestock species? The present study intends to answer the first question by comparing estimated relationships and inbreeding coefficients from three types of data: pedigree, SNP variants from the 50 K SNP chip and sequence variants from WGS data, as well as scenarios with different MAF restrictions. We focused our analysis on the effect of low MAF variants (below 5%) on estimated relationships and inbreeding coefficients.

## Methods

### Data

This study was performed on whole genome sequence and pedigree data from 118 Holstein bulls. All data used were already exiting and no animal experiments were involved. Of these 118, 63 originated from Europe (based on their Interbull IDs, 26 originated from the Netherlands, 12 from France, 11 from Denmark, 10 from Germany, two from Sweden, one from Finland and one from the United Kingdom), 19 from North-America (12 from the United States of America and seven from Canada) and 36 from Australia. They were selected as being important ancestors of the current Holstein populations in these countries. Pedigree records were available from the 1950s onwards and contained 4,054 individuals, 1,538 males and 2,516 females. The most represented sire had 53 offspring and the most represented dam had six. From the 118 bulls used for this study, 117 had birth date information and were born between 1968 and 2004. All 118 bulls had both parents recorded in the pedigree. From this group, 61 individuals were involved in a parent-offspring relationship (43 parent-offspring pairs). We counted two full sib pairs and 56 individuals were part of half-sib families containing two to five half-sibs. On average, individuals had partial pedigree records (missing dams or sires after generation one) of 13 generations and complete records of three generations (records for all dams and sires). A subgroup of 60 out of the 118 bulls had full pedigree records of at least two ancestral generations (full record on parent and grand-parent generations), of which 44 had full pedigree records at least up to four ancestral generations. These sub-groups were used for further analysis on inbreeding coefficients.

Whole genome sequence data for the selected bulls, including 28,336,153 SNPs (95% of the WGS variants) and 1,668,587 insertion-deletion variants (5% of the WGS variant) (hereafter jointly referred to as variants), were accessible through the 1000 bull genomes project (Run 3.0), and were for each individual obtained as described by Daetwyler et al. [11]. Sequencing was performed with Illumina HiSeq Systems (Illumina Inc., San Diego, CA). The procedure of editing the sequence data involved: sequence alignment, variant calling, phasing and quality controls. All called variants (SNPs and insertion-deletions) were put through an imputation step to fill any missing genotypes. The most likely genotypes after this imputation step were used in our study. SNPs that are included in the commonly used Illumina BovineSNP50 BeadChip v2 (Illumina Inc., San Diego, CA) were selected from the WGS, to enable computation of relationships based on SNP chip data. The average overall sequencing coverage was 10.5X (ranging from 3.2X to 38X), based on the 110 individuals for whom coverage information was available. Moreover, variants with a Minor Allele Frequency (MAF) lower than 1%, meaning that less than three copies of the minor allele were observed in the whole data set, were excluded from the analysis, as they may have represented genotyping errors. Note that using larger sample sizes may enable using lower MAF restriction thresholds. Out of the total number of sequenced variants present on the 29 autosomes, 18,739,233 on the WGS and 45,729 on the 50 K SNP chip were polymorphic in the 118 Holstein bulls. After applying the MAF quality control, i.e. remove variants with low MAF < 1%, 15,871,933 for WGS and 44,548 for the 50 K SNP chip were used for our analysis.

### Analysis of Hardy-Weinberg proportions

Hardy-Weinberg proportions analysis is traditionally performed as part of the editing process when using SNP data. In general, variants showing extreme departure from Hardy-Weinberg proportions are excluded from the analysis, as they are likely to represent genotyping errors. In our case we estimated the fraction of variants departing from Hardy-Weinberg proportions for each type of data and scenario of MAF restriction used in this study. The F-exact test was used to identify departure from Hardy-Weinberg proportions as it is the most suitable for cases of variants with low MAF [12]. For each segregating variant of the SNP and WGS data used in our study, *P*-*values* for the F-exact test were computed [13]. The fractions of variants departing from Hardy-Weinberg proportions, at a *P*-*value* ≤ 0.05 for the F-exact test, were calculated in each case.

### Relationship estimations

Additive genetic (**A**) and genomic (**G**) relationship matrices were computed. Two different methods were used to calculate the **G** matrix:

Firstly calculations were performed according to the Yang method [14] as follows:

$$ Gjk=\frac{1}{N}{\displaystyle \sum_i}Gijk=\left\{\begin{array}{c}\hfill \frac{1}{N}{\displaystyle \sum_i}\frac{\left(xij-2{p}_i\right)\left(xik-2{p}_i\right)}{2{p}_i\left(1-{p}_i\right)},\kern0.5em j\ \ne k\hfill \\ {}\hfill 1+\frac{1}{N}{\displaystyle \sum_i}\frac{{x_{ij}}^2-\left(1+2{p}_i\right){x}_{ij}+2{p_i}^2\ }{2{p}_i\left(1-{p}_i\right)},\kern0.5em j=k\hfill \end{array}\right. $$

Where *N* is the number of variants and *G*_*ijk*_ is the estimated relationship between individuals *j* and *k* at locus *i*. At each locus *i*, *x*_*i*._ is the individual variant genotype coded as 0, 1 or 2 and *p*_*i*_ is the frequency of the allele whose homozygote genotype is coded as 2 at locus *i*. Allele frequencies used in this case were estimated from the current population, as it is common practice in this type of analysis. The equation for *j* ≠ *k* is used to compute the off-diagonal elements of the **G** relationship matrix and the equation for *j*=*k* is used to compute the diagonal elements of the **G** relationship matrix.

Secondly, we computed relationships based on similarities by counting the number of identical alleles at segregating variants between individuals, which can be written as $\mathbf{G}=\frac{\left(\mathbf{M}-1\right){\left(\mathbf{M}-1\right)}^{\hbox{'}}}{\left(N/2\right)}$, where **M** is the genotype matrix containing values of 0, 1 and 2 and *N* is the number of variants. Derivation of the formula is explained in the Additional file 1.

According to Druet et al. [15], common variants have a MAF higher than 5% and MAF cut-off points ranging from 0.5% to 5% are commonly used as a lower MAF limit to remove variants in genetic studies [16]. In this study, we considered variants with a MAF below 5% to be variants with rare alleles. Relationships were computed for both estimators, using SNP (**G**_SNP_) and whole genome sequence data (**G**_WGS_) in three scenarios: (1) using all variants with a MAF higher than 5% (5+); (2) using all variants with a MAF higher than 1% (1+); (3) using variants with a MAF between 1% and 5% (1_5) in order to infer whether relationships based on variants with rare alleles were different from relationships based on common variants. After MAF restriction 41,225; 44,548 and 3,323 SNPs were kept for relationship estimation from the 50 K SNP chip (SNP), and 11,953,905; 15,871,933 and 3,918,028 from whole genome sequence (WGS) data, in scenario 5+, 1+ and 1_5, respectively (Table 1). Insertion-deletions represented 2.4%, 3.4% and 1% of the segregating variants in the three scenarios 5+, 1+ and 1_5.

**Table 1:** **Overview of the different scenarios**

**Scenario names**	**Type of data**	**Minor allele frequency threshold (%)**	**Number of segregating variants**
**A**_ped_	Pedigree	None	0
**G**_SNP_5+	BovineSNP50 BeadChip	≥ 5	41 225
**G**_SNP_1+	BovineSNP50 BeadChip	≥ 1	44 548
**G**_SNP_1_5	BovineSNP50 BeadChip	Between 1 and 5	3 323
**G**_WGS_5+	Whole genome sequence	≥ 5	11 953 905
**G**_WGS_1+	Whole genome sequence	≥ 1	15 871 933
**G**_WGS_1_5	Whole genome sequence	Between 1 and 5	3 918 028

### Comparison of estimated relationships between different scenarios

Estimated relationships using the three types of data (pedigree, SNP, and WGS) and the different scenarios (5+, 1+, and 1_5) were compared against each other. The relationships were split into groups and the cut-off points between these groups were defined according to pedigree estimated relationships as follows: self-relationships (relationships of the animal with itself), first degree relationships group such as parent-offspring or full sib relationships (relationships ≥0.5 to <1), second degree relationships group such as half sib, grandparents-offspring or cousin relationships (relationships ≥0.25 to <0.5) and less-related individuals (relationships <0.25) [17]. Only the three last groups were used for estimated relationship analysis, the first group (self-relationship group) was used for analysis of inbreeding. Differences between scenario 5+, 1+ and 1_5 were tested, using the Wilcoxon test, which is a non-parametric test of comparison of ranked sums between two paired groups [18]. Pearson’s correlation coefficients were computed between the different types of data: pedigree (**A**_ped_), and between SNP (**G**_SNP_) and WGS (**G**_WGS_) data with different MAF restriction scenarios in order to infer the impact of rare alleles on estimated relationships. All statistical analyses were conducted in R [19]. The test for correlation significance was performed using the R-package psych [20].

### Inbreeding coefficients

Inbreeding coefficients for pedigree were computed from the **A**_ped_ matrix using the algorithm of Sargolzaei et al. [21]. Genomic inbreeding coefficients were computed for each individual as the **G** matrix diagonal elements (self-relationship) minus 1. It should be noted that these inbreeding coefficients represent the correlation between uniting gametes in an individual [22]. Individuals were sub-grouped according to their pedigree depths: all 118 bulls had at least full pedigree records on their parents (group depth1); 60 of these 118 bulls had at least full pedigree records on two ancestral generations (group depth2) and finally, 44 had at least full pedigree records on four ancestral generations (group depth4). For inbreeding coefficients, correlations coefficients were computed between the different types of data with the different MAF restriction scenarios. All statistical analyses were conducted in R [19].

## Results

### Distribution of MAF and Hardy-Weinberg proportion analysis

**Figure 1:**
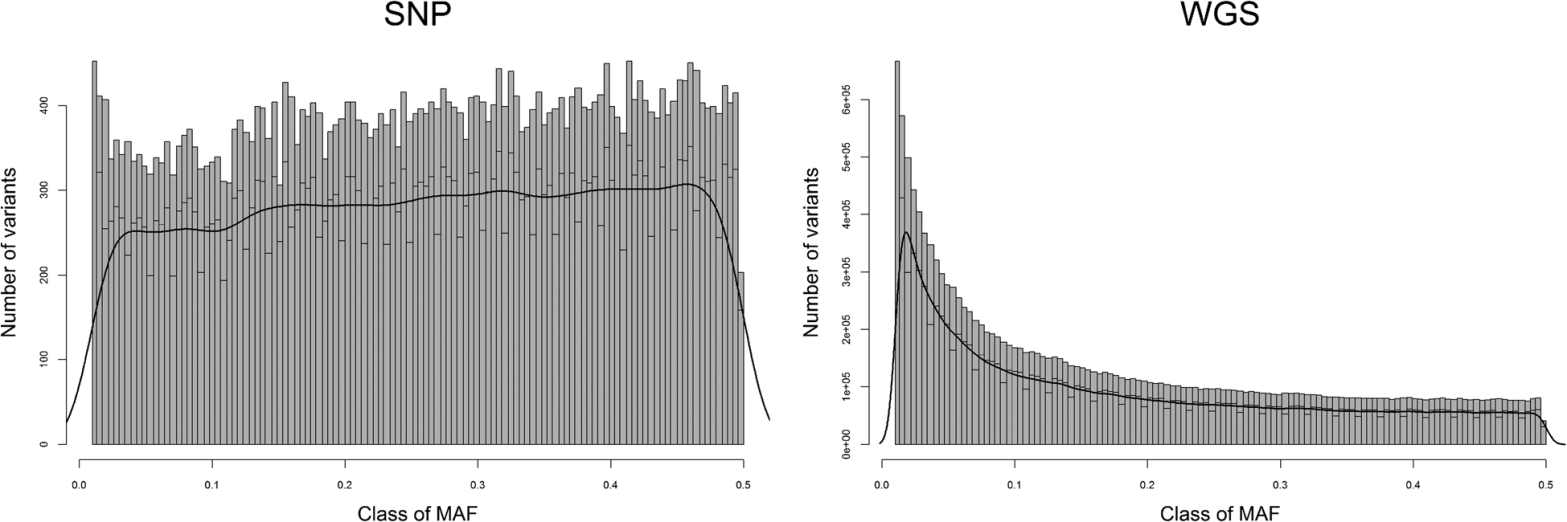
**Distribution plot of the number of variants per class of MAF.** Histograms of the number of segregating variants in each Minor Allele Frequency category (116 bins) from 1% to 50%, with density curve. The histogram on the left represents the distribution of variants from the Bovine 50 K SNP chip. The histogram on the right represents the distribution of variants from whole genome sequence (WGS) data.

A uniform distribution of MAF was observed for SNP variants, while a L shaped distribution was observed for sequence variants (Figure 1). As expected, all classes of MAF were equally represented on the SNP chip, while low MAF classes were overrepresented in sequence data. Scenarios including rare alleles (1_5 and 1+) showed a smaller fraction of departure from Hardy-Weinberg proportions (Table 2). This indicated that, contrary to our expectations, these scenarios were not more affected by departure from Hardy-Weinberg proportions than the other scenario based on common variants.

**Table 2:** **Hardy-Weinberg proportions analysis**

	**SNP5+**	**SNP1+**	**SNP1_5**	**WGS5+**	**WGS1+**	**WGS1_5**
Total variants	41 225	44 548	3 323	11 953 905	15 871 933	3 918 028
Departing variants	1 633	1 693	60	1 105 493	1 196 346	90 853
% departing variants	3.961	3.800	1.806	9.248	7.537	2.319

### Comparison of pedigree, SNP and sequence-based estimated relationships for common variants, MAF ≥ 5%

Estimated relationships for the three groups of different degrees of relationships (first, second and less-related) ranged from 0.00 to 0.66 for pedigree data, from –0.14 to 0.60 for SNP data and from –0.11 to 0.55 for WGS data (Table 3). Mean values for each considered degree of relationships were close to expectation for estimated relationships including deviations due to inbreeding. Variances of the SNP and WGS-based estimated relationships were in general higher than for pedigree estimated relationships for common variants, indicating that genomic data were able to capture more of the existing variance in relationships than pedigree data only.

**Table 3:** **Descriptive statistics (Yang method)**

	**Min**	**Mean**	**Max**	**Var**
First degree relationships				
**A**_ped_	0.503	0.548	0.663	0.0014
**G**_SNP_5+	0.368	0.464	0.603	0.0026
**G**_SNP_1+	0.355	0.453	0.617	0.0032
**G**_SNP_1_5	0.069	0.315	1.055	0.0367
**G**_WGS_5+	0.339	0.427	0.555	0.0023
**G**_WGS_1+	0.293	0.389	0.543	0.0033
**G**_WGS_1_5	0.128	0.275	0.692	0.0154
Second degree relationships				
**A**_ped_	0.250	0.302	0.406	0.0013
**G**_SNP_5+	0.100	0.216	0.440	0.0038
**G**_SNP_1+	0.094	0.209	0.445	0.0038
**G**_SNP_1_5	−0.022	0.113	0.517	0.0093
**G**_WGS_5+	0.075	0.200	0.402	0.0032
**G**_WGS_1+	0.059	0.177	0.382	0.0031
**G**_WGS_1_5	0.001	0.105	0.402	0.0048
Less-related				
**A**_ped_	0.000	0.056	0.245	0.0019
**G**_SNP_5+	−0.135	−0.015	0.382	0.0021
**G**_SNP_1+	−0.126	−0.015	0.386	0.0019
**G**_SNP_1_5	−0.112	−0.012	0.432	0.0011
**G**_WGS_5+	−0.113	−0.013	0.349	0.0018
**G**_WGS_1+	−0.092	−0.010	0.321	0.0013
**G**_WGS_1_5	−0.075	−0.001	0.599	0.0008
Inbreeding coefficients				
**A**_ped_	0.000	0.027	0.163	0.0009
**G**_SNP_5+	−0.244	−0.009	0.109	0.0023
**G**_SNP_1+	−0.234	−0.009	0.108	0.0021
**G**_SNP_1_5	−0.107	−0.014	0.176	0.0011
**G**_WGS_5+	−0.215	−0.037	0.068	0.0017
**G**_WGS_1+	−0.200	−0.060	0.045	0.0012
**G**_WGS_1_5	−0.273	−0.131	−0.021	0.0015

**Figure 2:**
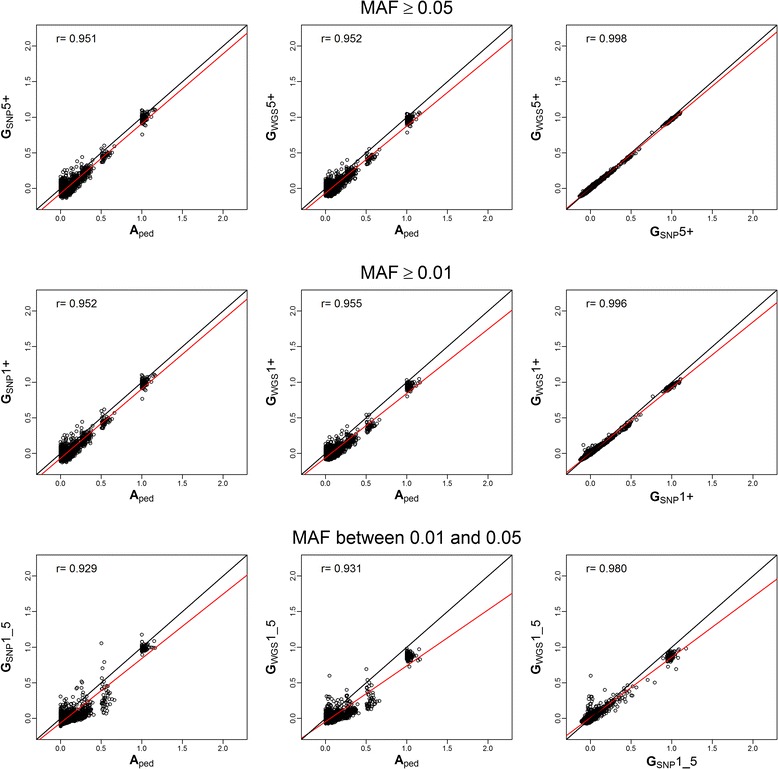
**Linear regressions plots for A** **,** **SNP and WGS against each other** **(** **Yang method** **)** **.** Plots of linear regressions of **A** estimated relationships from pedigree (**A**_ped_), **G** estimated relationships for Single Nucleotide Polymorphism (**G**_SNP_) and whole genome sequence (**G**_WGS_) data using the Yang method. Each linear regression was performed for the scenarios with Minor Allele Frequency (MAF) ≥ 5% (5+), ≥ 1% (1+) and between 1% and 5% (1_5). The first row represents the plots for scenario +5, the second for +1 and the third for 1_5. The first column shows the linear regression plots of **G**_SNP_ on **A**_ped_. The second column shows the linear regression plots of **G**_WGS_ on **A**_ped_. The third shows the linear regression plots of **G**_WGS_ on **G**_SNP_. In black is the regression line for an exact linear model (intercept=0, slope=1) and in red is the actual overall regression line. On the top left corner, the overall correlation coefficient for each linear regression appears.

Both **G**_SNP_ and **G**_WGS_ had a correlation of 0.95 with **A**_ped_, while **G**_SNP_ and **G**_WGS_ had a correlation of 0.99 (Figure 2). Correlations across all relationships were higher than correlations within groups of relationships (Table 4). In fact, correlations across all relationships indicated that groups of relationships were ranked similarly, as expected, when computed from different data. However, correlations within groups showed that using pedigree or genetic variants yielded quite different individual estimated relationships. Correlation coefficients between **A**_ped_ and **G** were moderate (ranging from 0.36 to 0.51; Table 4). Correlations between **G**_SNP_ and **G**_WGS_ were similarly high for the three relationship groups (0.98).

**Table 4:** **Correlation coefficients for estimated relationships and inbreeding coefficients (Yang method)**

	**Estimated relationships**			**Inbreeding coefficients**		
	**First degree**	**Second degree**	**Less-related**	**Depth1**	**Depth2**	**Depth4**
**A**_ped_ ~ **G**_SNP_5+	0.450^a,b^	0.372^a,b^	0.511^a,b^	0.395^a,b^	0.595^a,b^	0.721^a,b^
**A**_ped_ ~ **G**_WGS_5+	0.487^a,b^	0.361^a,b^	0.512^a,b^	0.392^a,b^	0.579^a,b^	0.710^a,b^
**G**_WGS_5+ ~ **G**_SNP_5+	0.973^a,b^	0.982^a,b^	0.979^a,b^	0.979^a,b^	0.985^a,b^	0.985^a,b^
**A**_ped_ ~ **G**_SNP_1+	0.335^a,b^	0.351^a,b^	0.516^a,b^	0.391^a,b^	0.601^a,b^	0.723^a,b^
**A**_ped_ ~ **G**_WGS_1+	0.212^b^	0.286^a,b^	0.514^a,b^	0.360^a,b^	0.570^a,b^	0.689^a,b^
**G**_WGS_1+ ~ **G**_SNP_1+	0.948^a,b^	0.967^a,b^	0.966^a,b^	0.933^a,b^	0.936^a,b^	0.946^a,b^
**A**_ped_ ~ **G**_SNP_1_5	−0.162^b^	0.045^b^	0.374^a,b^	0.122^b^	0.448^a,b^	0.501^a,b^
**A**_ped_ ~ **G**_WGS_1_5	−0.170^b^	0.022^b^	0.351^a,b^	0.035^b^	0.142^b^	0.198^b^
**G**_WGS_1_5 ~ **G**_SNP_1_5	0.950 ^a,b^	0.857^a,b^	0.676^a,b^	0.515^a,b^	0.487^a,b^	0.537^a,b^
**G**_SNP_1+ ~ **G**_SNP_5+	0.978^a,b^	0.995^a^	0.999^a^	0.999^a^	0.999^a^	0.999^a^
**G**_WGS_1+ ~ **G**_WGS_5+	0.888^a,b^	0.972^a,b^	0.989^a,b^	0.965^a,b^	0.969^a,b^	0.978^a,b^
**G**_SNP_5+ ~ **G**_SNP_1_5	0.567^a,b^	0.587^a,b^	0.555^a,b^	0.446^a,b^	0.467^a,b^	0.588^a,b^
**G**_WGS_5+ ~ **G**_WGS_1_5	0.503^a,b^	0.647^a,b^	0.600^a,b^	0.263^a,b^	0.185^b^	0.315^a,b^
**G**_SNP_1+ ~ **G**_SNP_1_5	0.725^a,b^	0.661^a,b^	0.593^a,b^	0.488^a,b^	0.494^a,b^	0.611^a,b^
**G**_WGS_1+ ~ **G**_WGS_1_5	0.844^a,b^	0.808^a,b^	0.714^a,b^	0.507^a,b^	0.423^a,b^	0.505^a,b^

^a,b^where ^a^means significantly different from 0 and ^b^significantly different from 1 (*P*-*value* ≤0.05).

Inbreeding coefficients were on average close to zero for SNP and WGS, ranging from 0 to 0.16 for pedigree estimates, from –0.24 to 0.11 for SNP and from –0.21 to 0.07 for WGS. Correlations between pedigree and genomic inbreeding increased with pedigree depth, as expected. Significant differences between correlations were observed between depth1 and depth4, for **A**_ped_ versus **G**_SNP_5+ or **G**_WGS_5+ (*P*-*value*=0.01).

### Comparison of pedigree, SNP and sequence-based estimated relationships when including rare alleles

Estimated relationships for scenario 1+ and 1_5 varied from slightly negative (−0.13) for the less related group to highly positive (1.06) for first degree relationships group (Table 3). Mean values within groups of different degrees of relationships ranged between 0.45 and 0.27 for the first degree relationships group, between 0.21 and 0.10 for the second degree relationships group and between 0 and –0.01 for the less-related group, i.e. slightly lower than the theoretical expectations. Variances were in general larger for SNP than for WGS.

When comparing scenarios including rare alleles, we observed that the correlations between **A**_ped_ and **G** estimated relationships were in general lower than for scenario 5+. Very low correlations were observed between **A**_ped_ and **G** for scenario 1_5 with most of the correlations being non-significantly different from zero. High correlations between **G**_SNP_ and **G**_WGS_ data were observed for scenario 1+ (on average 0.96) and scenario 1_5 (on average 0.83); both being lower than the value of 0.98 observed for 5 +.

Inbreeding coefficients ranged from –0.23 to 0.18 for SNP and from –0.27 to 0.04 for WGS across the two scenarios including rare alleles. Correlations between pedigree and genomic inbreeding coefficients increased with pedigree depth. Difference in correlations was significant between depth1 and depth4 when comparing **G**_SNP_1+ and **G**_WGS_1+ to **A**_ped_ (*P*-*value*=0.01), and between depth1 and other depths for **G**_SNP_1_5 compared to **A**_ped_ (*P*-*value*=0.02). Similar as for the relationships, scenario 1_5 showed important differences with scenario 1+ as correlations between **A**_ped_ and **G**_SNP_1_5 for depth1 and all between **A**_ped_ and **G**_WGS_1_5 were not significantly different from zero.

### Estimated relationships and inbreeding coefficients based on common versus rare alleles

Hereafter we report correlations within **G**_SNP_ and **G**_WGS_, between the different MAF scenarios (e.g. between **G**_SNP_5+ and **G**_SNP_1+, **G**_SNP_5+ and **G**_SNP_1_5 or **G**_SNP_1+ and **G**_SNP_1_5) (Table 4). Comparative Wilcoxon tests showed significant differences between the estimated relationships of the different scenarios (*P*-*value* <1.10^−6^). Regarding inbreeding coefficients, differences between scenarios were only significant when computed from whole genome sequence data (*P*-*value* <1.10^−6^). Correlation between scenario 1+ and 5+ for **G**_SNP_, in almost all group of degrees of relationships, did not show significant difference from 1, adding variants with low MAF did not affect estimated relationships when using SNP. As scenario 1_5 and 1+ partly used the same variants, they were, for both **G**_WGS_ and **G**_SNP_, better correlated (0.84 to 0.59) than 1_5 and 5+ (0.65 to 0.50). Moreover, the correlations between scenario 1+ and 1_5 for **G**_WGS_ were higher than for **G**_SNP_, indicating that the exclusive use of variants with a MAF between 1% and 5% gave estimates that were closer to the estimated relationships of WGS data, as the latter type of data contains relatively more of these variants.

### Similarity-based estimated relationships

Alongside the Yang method, which weighs the contribution of each locus by its MAF, we also computed relationships based on similarities between genotypes. This yielded estimated relationships that were generally higher and with smaller variances than those yielded by the Yang method. Estimated relationships for genomic data ranged from 0.40 to 1.94; in particular scenario 1_5 showed high genomic estimated relationships ranging from 1.47 to 1.94 (Table 5). In fact, relationships estimated using the method based on similarities are expected to fall in the range from –2 to 2, –2 corresponding to two individuals having opposing homozygote genotypes for all variants and 2 denoting identical homozygote genotypes for all variants. The scenario including only variants with rare alleles showed estimates close to 2. This can be explained by the fact that variants with low MAF in the current population harboured a high proportion of homozygous individuals for the common allele, compared to individuals being heterozygous or homozygous for the minor allele. Indeed, individuals are likely to be more similar for the common allele when looking at low MAF variants, causing by construction higher values for scenario 1_5.

**Table 5:** **Descriptive statistics (based on similarities)**

	**Min**	**Mean**	**Max**	**Var**
First degree relationships				
**A**_ped_	0.503	0.548	0.663	0.0014
**G**_SNP_5+	0.815	0.876	0.974	0.0011
**G**_SNP_1+	0.891	0.949	1.040	0.0010
**G**_SNP_1_5	1.686	1.851	1.939	0.0026
**G**_WGS_5+	0.957	1.008	1.080	0.0006
**G**_WGS_1+	1.165	1.209	1.265	0.0005
**G**_WGS_1_5	1.719	1.822	1.876	0.0013
Second degree relationships				
**A**_ped_	0.250	0.302	0.407	0.0013
**G**_SNP_5+	0.617	0.693	0.847	0.0021
**G**_SNP_1+	0.705	0.778	0.921	0.0019
**G**_SNP_1_5	1.622	1.830	1.910	0.0028
**G**_WGS_5+	0.786	0.864	1.009	0.0013
**G**_WGS_1+	1.034	1.096	1.207	0.0009
**G**_WGS_1_5	1.661	1.807	1.859	0.0016
Less-related				
**A**_ped_	0.000	0.056	0.245	0.0019
**G**_SNP_5+	0.405	0.502	0.746	0.0017
**G**_SNP_1+	0.501	0.597	0.829	0.0017
**G**_SNP_1_5	1.477	1.773	1.925	0.0040
**G**_WGS_5+	0.634	0.715	0.911	0.0010
**G**_WGS_1+	0.889	0.976	1.132	0.0009
**G**_WGS_1_5	1.576	1.771	1.868	0.0017
Inbreeding coefficients				
**A**_ped_	0.000	0.027	0.163	0.0009
**G**_SNP_5+	0.003	0.251	0.347	0.0015
**G**_SNP_1+	0.059	0.298	0.390	0.0014
**G**_SNP_1_5	0.706	0.886	0.974	0.0020
**G**_WGS_5+	0.163	0.342	0.417	0.0010
**G**_WGS_1+	0.321	0.473	0.537	0.0007
**G**_WGS_1_5	0.764	0.873	0.930	0.0009

**Figure 3:**
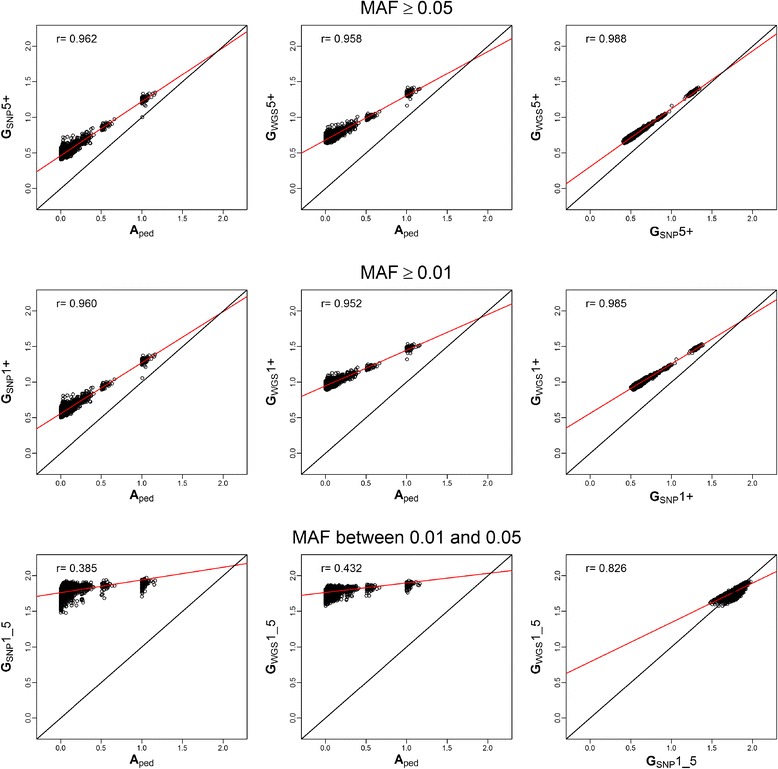
**Linear regressions plots for A, SNP and WGS against each other (based on similarities).** Plots of linear regression of **A** estimated relationships from pedigree (**A**_ped_), **G** estimated relationships for Single Nucleotide Polymorphism (**G**_SNP_) and whole genome sequence (**G**_WGS_) data, based on similarities. Each linear regression was performed for the scenarios with Minor Allele Frequency (MAF) ≥ 5% (5+), ≥ 1% (1+) and between 1% and 5% (1_5). The first row represents the plots for scenario +5, the second for +1 and the third for 1_5. The first column shows the linear regression plots of **G**_SNP_ on **A**_ped_. The second column shows the linear regression plots of **G**_WGS_ on **A**_ped_. The third shows the linear regression plots of **G**_WGS_ on **G**_SNP_. In black is the regression line for an exact linear model (intercept=0, slope=1) and in red is the actual overall regression line. On the top left corner, the overall correlation coefficient for each linear regression appears.

Overall, correlations from the similarity-based method and Yang method were similar between **A**_ped_ and **G** estimated relationships for scenarios 5+ and 1+ (0.96). The overall correlations between the **A**_ped_ and **G** in scenario 1_5 were smaller for similarities, which where 0.43 for **G**_WGS_ and 0.39 for **G**_SNP_ (Figure 3); for the Yang method, results were 0.93 for **G**_WGS_ and for **G**_SNP_ (Figure 2). The major difference observed when using the similarity-based method instead of the Yang method was that correlations between **A**_ped_ and **G**_SNP_ or **G**_WGS_, within groups of different degrees of relationships, were noticeably higher. On the other hand, when comparing scenario 1+ and 5+ to 1_5 for both **G**_SNP_ and **G**_WGS_, correlations based on similarities were smaller (Table 6).

**Table 6:** **Correlation coefficient for estimated relationships and inbreeding coefficients (based on similarities)**

	**Estimated relationships**			**Inbreeding coefficients**		
	**First degree**	**Second degree**	**Less-related**	**Depth1**	**Depth2**	**Depth4**
**A**_ped_ ~ **G**_SNP_5+	0.703^a,b^	0.531^a,b^	0.698^a,b^	0.474^a,b^	0.618^a,b^	0.665^a,b^
**A**_ped_ ~ **G**_WGS_5+	0.618^a,b^	0.508^a,b^	0.633^a,b^	0.394^a,b^	0.544^a,b^	0.616^a,b^
**G**_WGS_5+ ~ **G**_SNP_5+	0.936^a,b^	0.935^a,b^	0.916^a,b^	0.928^a,b^	0.950^a,b^	0.962^a,b^
**A**_ped_ ~ **G**_SNP_1+	0.700^a,b^	0.542^a,b^	0.707^a,b^	0.484^a,b^	0.622^a,b^	0.660^a,b^
**A**_ped_ ~ **G**_WGS_1+	0.610^a,b^	0.551^a,b^	0.660^a,b^	0.425^a,b^	0.565^a,b^	0.601^a,b^
**G**_WGS_1+ ~ **G**_SNP_1+	0.915^a,b^	0.909^a,b^	0.905^a,b^	0.914^a,b^	0.934^a,b^	0.947^a,b^
**A**_ped_ ~ **G**_SNP_1_5	0.259^b^	0.286^a,b^	0.474^a,b^	0.269^a,b^	0.269^a,b^	0.237^b^
**A**_ped_ ~ **G**_WGS_1_5	0.222^b^	0.277^a,b^	0.423^a,b^	0.242^a,b^	0.248^b^	0.201^b^
**G**_WGS_1_5 ~ **G**_SNP_1_5	0.869^a,b^	0.791^a,b^	0.813^a,b^	0.782^a,b^	0.697^a,b^	0.666^a,b^
**G**_SNP_1+ ~ **G**_SNP_5+	0.994^a^	0.996^a^	0.995^a^	0.996^a^	0.998^a^	0.999^a^
**G**_WGS_1+ ~ **G**_WGS_5+	0.922^a,b^	0.947^a,b^	0.949^a,b^	0.960^a,b^	0.970^a,b^	0.983^a,b^
**G**_SNP_5+ ~ **G**_SNP_1_5	0.346^a,b^	0.260^a,b^	0.521^a,b^	0.280^a,b^	0.307^a,b^	0.508^a,b^
**G**_WGS_5+ ~ **G**_WGS_1_5	0.194^b^	0.115^b^	0.398^a,b^	0.195^a,b^	0.185^b^	0.367^a,b^
**G**_SNP_1+ ~ **G**_SNP_1_5	0.449^a,b^	0.343^a,b^	0.603^a,b^	0.362^a,b^	0.365 ^a,b^	0.543^a,b^
**G**_WGS_1+ ~ **G**_WGS_1_5	0.559^a,b^	0.427^a,b^	0.668^a,b^	0.462^a,b^	0.417^a,b^	0.533^a,b^

^a,b^where^ a^means significantly different from 0 and ^b^significantly different from 1 (*P*-*value* ≤0.05).

Correlations between inbreeding coefficients obtained from different data sets when using similarities were mostly not significantly different than those yielded by the Yang method (Table 6). Inbreeding coefficients from pedigree were on average close to zero, for SNP and WGS, in both scenarios 5+ and 1+, around 0.35 and even higher (0.88) for the scenario 1_5, due to using a value of 0.5 for all allele frequencies.

## Discussion

Whole genome sequence data cover all SNP and structural variation and are therefore expected to estimate exact relationships between individuals. With the increasing availability of this source of information, one major question is whether relationships estimated from whole genome sequence data are indeed different from those computed from pedigree and SNP data, and whether such differences justify the replacement of traditional data by WGS information. Pérez-Enciso [23] suggested that new generation sequencing techniques are as valuable as high density SNP chips for estimating genomic relationships, provided that coverage and variant density of SNP chips are sufficient. However, an important benefit of using WGS instead of pedigree and SNP data is that it enables access, without any ascertainment bias, to information on all variants with rare alleles. Variants with a MAF between 1% and 5%, defined here as variants with rare alleles, represented approximately 20% of the segregating variants of the WGS in our study, a relatively large proportion of the whole genome sequence variants, but only 7% of the SNP data. In this study, we showed that additional information from rare alleles can have a significant impact on estimated relationships and (to a lesser extent) on inbreeding coefficients. Since these estimates provide the basis for selection decisions, it can be hypothesised that using sequence data instead of SNP data will affect subsequent selection and that including rare variants in the data used for estimation will allow focusing more on the variation carried by such rare variants.

### Whole genome sequence data

Whole genome sequencing is a rapidly developing field, making new tools available for animal breeding but some limitations are still to be reported. One issue with WGS is the variant calling accuracy, that tends to be low at variants showing extreme minor allele frequencies [24]. The current approach taken for WGS in cattle, is to sequence key ancestors in the population [11], and then impute this sequence data for other animals in the population that are genotyped with high density SNP chips [24]. Results of imputation of WGS show poor accuracy for variants with low MAF of 5% and lower, the accuracy of imputation decreases to below 0.5 [11]. Pérez-Enciso [23] argued that high density SNP chips are cheaper and more reliable than data from sequencing followed by imputation. The issue of low imputation accuracy may be overcome by using a larger sample size [15]. Further investigations and applications of whole genome sequence data are expected to benefit from the growing number of available sequences, and the development of better imputation strategies [15,25].

Accuracy of the estimated allele frequencies may affect estimated relationships, in the sense that small sample sizes might lead to increased estimation error. To asses the impact of this issue on our results we performed a simulation study (details in Additional file 2). Allele frequencies, for each variant of the WGS selected in scenario 1+, were drawn 100 times from a normal distribution with mean and variance measured from the observed allele frequencies. Using each of the 100 sets of simulated allele frequencies, we computed the relationships with the Yang method, and correlated them with the estimated relationships using the observed allele frequencies. These correlations were all greater than 0.999, showing that our results were not affected by innaccuracy of estimated allele frequencies due to limited sample size.

Finally, in addition to our analysis of the complete WGS variants set, we performed the relationship computations excluding insertion-deletion variants. Correlations between estimates from all variants or excluding insertion-deletions were equal to 1 (results not shown). This observation supported our conclusion that changes between scenarios and type of data were due to low MAF variants, and not because the sequence data also included insertion-deletion variants.

### Relationship estimators

Differences between pedigree and marker-based estimators have three main causes. Firstly, pedigree estimators rely on the fact that 50% of the genome is transmitted from parents to offspring. Likewise, two non-inbred full sibs theoretically are expected to share 50% of their genome. Marker-based methods, however, give access to the actual shared proportion. In the case of full sibs, for example, the share of genome might vary from the 50% value due to Mendelian sampling [26]. Secondly, pedigree-based methods assume that individuals with unknown parents do not have alleles in common. Therefore, pedigree-based estimators measure the proportion of genome shared by two individuals descending from an assumed unrelated founder population; Identical By Descent (IBD). Marker-based methods, on the other hand, estimate the proportion of the genome that is Identical By State (IBS). Marker-based estimators, such as the Yang method, apply correction for allele frequencies that increases the weight of low MAF variants. Such estimators are therefore expected to be more similar to IBD estimators, relative to the base population from which the allele frequencies are defined. Finally, the estimators differ in the way that this base population is assigned. Pedigree estimators assume an arbitrary base population, defined as the founder individuals in the pedigree. Marker-based estimators define the base populations depending on the allele frequencies used for the estimation. The similarity-based method is defined as being an estimator of relationships when founder alleles are unique [27]. It is equivalent to defining the founder population further back in time, as confirmed by the high inbreeding coefficients obtained in this study. As argued by VanRaden [28], estimated relationships should be computed using allele frequencies from the founder population. Since the actual founder population is usually unknown, these estimates may be computed from the base population in the pedigree. One way to do this is described by Gengler et al. [29]. In practice, due to difficulties for coping with discrepancies in pedigree completeness and depth, allele frequencies from the current population are mostly used. Likely because such frequencies had been used to compute the Yang estimator in our study, the considered base population when computing similarities was closer to the base population of the pedigree than to the one used in the Yang estimator. Evidence can be seen in our results; more similar relationships, so higher correlations, were observed between pedigree-based and similarity-based estimators than between pedigree-based and the Yang estimator. As suggested by Luan et al. [30], different estimators capture different ages of relationships and when the earliest relationships are of interest, IBS estimators will be more accurate than estimators based on pedigree.

Analogous to our similarity-based method, Pérez-Enciso [23], in a simulation study, estimated relationships based on the fraction of alleles shared by two individuals without accounting for differences in allele frequencies. Forni et al. [31] also compared different scenarios based on similarities, or allele frequencies when using SNP data. Both Forni et al. [31] and Pérez-Enciso [23] argued that the use of estimators scaled by the allele frequencies, such as achieved by the Yang estimator used in our study, provide standardised diagonal and off-diagonal estimates, which are more appropriate for further application in selection strategies.

By correcting for allele frequencies, the Yang estimator puts relatively more emphasis on low MAF variants. Rare alleles are either recent mutations or ancient alleles driven to low allele frequencies through time due to drift, or natural and artificial selection. These alleles have a higher risk for disappearing after a few generations; thus in the framework of genetic diversity conservation, it may be desirable to put a higher priority on rare compared to common alleles in order to balance the potential loss of genetic diversity. This suggests that the Yang estimator may also be most appropriate when computed relationships are used for genetic diversity conservation decisions, which aim to conserve variation at low MAF variants as much as possible.

### Comparison of pedigree, SNP and sequence-based standardised estimates

In our study, correlations were high only between **G**_SNP_ and **G**_WGS_ (ranging from 0.68 to 0.98 for all scenarios), in agreement with a correlation of 0.92 between both scenarios reported by Pérez-Enciso [23]. Additionally, in our study, the correlation between **G**_SNP_ and **G**_WGS_ on one hand and **A** on the other hand were considerably lower and variances of estimated relationships were generally higher for both **G**_SNP_ and **G**_WGS_ than for **A**, comparable to results found in other studies [31-34].

Grouping individuals according to their pedigree depths showed that longer pedigree records led to closer correlation between pedigree and genomic inbreeding coefficients. Negative inbreeding coefficients, i.e. self-relationships lower than one, were also observed. With ‘inbreeding’ defined as the mating of individuals that are more related than the average of the population [34], negative inbreeding coefficients occur when individuals have an excess of observed heterozygous genotypes, compared to the expected number based on the allele frequencies of the population [35]. Finally, in this study we observed that inbreeding coefficients computed from whole genome sequence data were significantly different depending on the MAF restriction chosen.

Pérez-Enciso [23] argued that relaxing the MAF cut-off point for variants array design, which are customised according to a population, can be used for more accurate relationship estimation. Edriss et al. [16] also argue that a MAF restriction between 0.01 and 0.02, instead of a higher threshold, may lead to an improvement in the accuracy of genomic predictions. Rare alleles are of interest in genetic diversity conservation. From our results it can be speculated that including variant with low MAF, by using WGS information, may impact prioritisation for genetic diversity conservation. Further studies are needed to confirm this hypothesis.

## Conclusions

Relationships computed from whole genome sequence data are expected to reflect the true relationships between individuals; therefore, sequence data are considered a valuable resource for improving estimated relationships. In this study, estimated relationships and inbreeding coefficients from pedigree and genomic information were hardly correlated; when from SNP and WGS data they were shown to be strongly correlated. Nevertheless, when using the sequence data, neglecting rare alleles, i.e. variants with a MAF below 5%, led to significant changes in the estimated relationships. Such changes may affect selection strategies for long-term selection and genetic diversity conservation. If conservation of genetic diversity is geared towards safeguarding all accessible variation, then relationship estimators that weigh genotypes by their allele frequencies are to be preferred, possibly combined with the use of sequence data. The following question, however, remains un-answered: to what extent will the use of whole genome sequence data and rare allele information affect selection strategies such as Optimal Contribution Selection in optimising long-term genetic improvement and genetic diversity conservation?

## Additional files

Additional file 1:
**G matrix computation based on similarities.** Derivation of the formula used for similarity-based G matrix computation. Additional file 2:
**Sampling error of allele frequency estimation.** Simulation study of allele frequency estimation and assessment of sampling error.

## Abbreviations

MAF: Minor allele frequency
SNP: Single nucleotide polymorphism
WGS: Whole genome sequence
A: Additive relationship matrix
G: Genomic relationship matrix
IBD: Identity by descent
IBS: Identity by state
