# Supplementary material for: The effect of rare alleles on estimated genomic relationships from whole genome sequence data
Source: BMC Genet. 2015 Mar 12;16:24. doi: 10.1186/s12863-015-0185-0 (PMC4365517; doi:10.1186/s12863-015-0185-0)
Supplement: Additional file 1: — G matrix computation based on similarities. Derivation of the formula used for similarity-based G matrix computation. [file 12863_2015_185_MOESM1_ESM.pdf]

Additional file 1 - G matrix computation based on similarities

Similarity (S) can be measured as the number of shared alleles between individuals *j* and *k* for each genotype at locus *i*, such as [1]

$$S_{jk}, i = \frac{I_{j1k1} + I_{j1k2} + I_{j2k1} + I_{j2k2}}{4}$$

For a single locus, S_{jk} =

ind_j / ind_k	AA	AB	BB
**AA**	1	0.5	0
**AB**	0.5	0.5	0.5
**BB**	0	0.5	1

We shall now show that this is similar to computing relationships using the following equations as outlined by Yang et al [2], with allele frequency p_i fixed at 0.5 for all variants.

$$G_{jk} = \frac{1}{N}\sum_i G_{ijk} = \begin{cases} \frac{1}{N}\sum_i \frac{(x_{ij} - 2p_i)(x_{ik} - 2p_i)}{2p_i(1 - p_i)}, & j \neq k \quad (\mathbf{1}) \\ 1 + \frac{1}{N}\sum_i \frac{x_{ij}^2 - (1 + 2p_i)x_{ij} + 2p_i^2}{2p_i(1 - p_i)}, & j = k \quad (\mathbf{2}) \end{cases}$$

Let's consider a unique locus,

$$(\mathbf{1}) \qquad \frac{(x_{ij} - 2p_i)(x_{ik} - 2p_i)}{2p_i(1 - p_i)} = \frac{(x_{ij} - 1)(x_{ik} - 1)}{0.5}$$

$$(\mathbf{2}) \qquad 1 + \frac{x_{ij}^2 - (1 + 2p_i)x_{ij} + 2p_i^2}{2p_i(1 - p_i)} = 1 + \frac{x_{ij}^2 - 2x_{ij} + 0.5}{0.5}$$

$$= \frac{x_{ij}^2 - 2x_{ij} + 1}{0.5} = \frac{(x_{ij} - 1)(x_{ij} - 1)}{0.5}$$

If $x_{ij} = x_{ik}$ then **(1)** = **(2)** and only one equation is needed to calculate both diagonal and off-diagonal elements.

In this case, G_{jk} =

ind_j / ind_k	AA [2]	AB [1]	BB [0]
**AA [2]**	2	0	-2
**AB [1]**	0	0	0
**BB [0]**	-2	0	2

S and *G* are linked by the following transformation: $4 * \left(S_{jk} - \overline{S_{jk}}\right) = G_{jk}$, with $\overline{S_{jk}}$ = 0.5.

In the case of multiple loci,

$$G_{jk} = \frac{1}{N}\sum_i G_{ijk} = \frac{1}{N}\sum_i \frac{(x_{ij} - 1)(x_{ik} - 1)}{0.5}$$

Which is equivalent, in matrix notation, to $\mathbf{G} = \frac{(\mathbf{M}-\mathbf{1})(\mathbf{M}-\mathbf{1})'}{(N/2)}$, where **M** is the genotype matrix containing values of 0, 1 and 2.

1. Eding H, Crooijmans R, Groenen MAM, Meuwissen THE: **Assessing the contribution of breeds to genetic diversity in conservation schemes**. *Genet Sel Evol* 2002, **34**(5):613-633.
2. Yang JA, Benyamin B, McEvoy BP, Gordon S, Henders AK, Nyholt DR, Madden PA, Heath AC, Martin NG, Montgomery GW, Goddard ME, Visscher PM: **Common SNPs explain a large proportion of the heritability for human height**. *Nat Genet* 2010, **42**(7):565-569.
