# Supplementary material for: The effect of rare alleles on estimated genomic relationships from whole genome sequence data
Source: BMC Genet. 2015 Mar 12;16:24. doi: 10.1186/s12863-015-0185-0 (PMC4365517; doi:10.1186/s12863-015-0185-0)
Supplement: Additional file 2: — Sampling error of allele frequency estimation. Simulation study of allele frequency estimation and assessment of sampling error. [file 12863_2015_185_MOESM2_ESM.pdf]

Additional file 2 – Sampling error of allele frequency estimation

Methods

The sampling variance of the estimate of allele frequency (p) can be calculated as

$$var(p) = \frac{p * (1 - p)}{2N}$$

where p and (1-p) are the observed allele frequency at one diploid marker and N is the number of individuals in the population from which we estimated the allele frequency p. Such estimate was calculated independently for each of the 15,871,933 variants used in the scenario 1+. For each variant we randomly sampled, 100 times, a simulated allele frequency from a Normal distribution $N(\mu, \sigma)$ with a mean $\mu = p$ and a standard deviation $\sigma = \sqrt{var(p)}$, for the 15,871,933 variants. Thereafter these allele frequencies were used to compute estimated relationships using the Yang method [1], with a minor allele frequency restriction at 1% (scenario 1+). So, variants that had a sampled MAF below 1% were not included. Finally, we compared the estimated relationships from the observed allele frequencies with the ones from the simulated allele frequencies by calculating correlation coefficients using R [2].

Results

Correlations between estimated relationships from the observed allele frequencies and estimated relationships from the simulated allele frequencies ranged from 0.999810 to 0.999813 with an average of 0.999812.

1. Yang JA, Benyamin B, McEvoy BP, Gordon S, Henders AK, Nyholt DR, Madden PA, Heath AC, Martin NG, Montgomery GW, Goddard ME, Visscher PM: **Common SNPs explain a large proportion of the heritability for human height**. *Nat Genet* 2010, **42**(7):565-569.
2. R Development Core Team: **R: A language and environment for statistical computing**. In.: R Foundation for Statistical Computing; 2011.
